# Piperine-loaded solid lipid nanoparticles: a promising nano-phytomedicine for the treatment of non-alcoholic fatty liver disease

**DOI:** 10.3389/fphar.2025.1646530

**Published:** 2026-01-14

**Authors:** Afrasim Moin, Shwetha Ram, Sateesha Shivally Boregowda, Talib Hussain, Amr Selim Abu Lila, Rajamma Abburu Jayaramu, Syed Mohd Danish Rizvi, Sirajudheen Anwar, Kalegowda Chandan

**Affiliations:** 1 Department of Pharmaceutics, College of Pharmacy, University of Hail, Hail, Saudi Arabia; 2 Department of Pharmaceutics, Acharya & BM Reddy College of Pharmacy, Bengaluru, India; 3 Department of Pharmacology and Toxicology, College of Pharmacy, University of Hail, Hail, Saudi Arabia; 4 Department of Pharmacognosy, KLE College of Pharmacy, Bengaluru, KLE Academy of Higher Education and Research, Belagavi, Karnataka, India; 5 Department of Postharvest Management, University of Horticultural Sciences, Bagalkot, India

**Keywords:** liver enzymes, non-alcoholic fatty liver disease, oral glucose tolerance test, piperine, solid lipid nanoparticles

## Abstract

**Introduction:**

Piperine (PIP), the active alkaloid found in black pepper (*Piper nigrum*), has gained attention for its potential therapeutic role in managing non-alcoholic fatty liver disease (NAFLD) due to its anti-inflammatory, lipid-lowering, antioxidant, and insulin-sensitising properties. Nevertheless, PIP’s poor solubility limits its absorption in the gastrointestinal tract, thereby, compromising its systemic bioavailability. Consequently, the objective of this research has been to formulate piperine-loaded solid lipid nanoparticles (PIP- SLNs) so as to increase its oral bioavailability and prolong its hepatic circulation time.

**Methods:**

Herein, PIP-SLNs were prepared by the hot homogenization method. The fabricated PIP-SLNs were characterized for size, zeta potential, surface morphology, entrapment efficiency and *in vitro* release performance. The impact of the optimized formula (F6) on key parameters associated with NAFLD, such as oral glucose tolerance (OGTT), aspartate aminotransferase (AST), alanine aminotransferase (ALT), total cholesterol (TC), triglyceride (TG) levels, and liver weight, was evaluated using a hyperlipidaemic Swiss albino mice model. Additionally, liver histopathology was examined pre- and post-treatment to assess the efficacy of PIP-SLNs in mitigating hyperlipidaemia.

**Results and discussion:**

Particle size, zeta potential, and drug entrapment efficiency of the optimized formula (F6) was found to be 191.2 ± 27.9 nm, - 20 ± 1.3 mV, 72.3% ± 2.8, respectively. Morphologically, the PIP-SLNs were found to be spherical. The optimized formulation (F6) exhibited sustained release up to 70% at 48 h, fitting the Higuchi model (R^2^ = 0.976) indicative of diffusion-driven release, with a Korsmeyer‐Peppas exponent (n = 0.63), further confirming anomalous diffusion-relaxation transport. Most importantly, the NAFLD study demonstrated a significant (p < 0.05) drop in blood glucose levels, serum markers (AST and ALT, p < 0.001), total cholesterol and triglycerides (p < 0.05), and also liver weight (p < 0.028), which was far superior to those elicited by plain PIP suspension. These findings reiterate the potential of solid lipid nanoparticles in increasing the bioavailability and thereby its hepatic circulation of PIP, which in turn, significantly enhanced its hepatoprotective effect in NAFLD.

## 1 Introduction

The liver, the body’s largest and most metabolically active organ, plays a critical role in maintaining homeostasis by regulating lipid, carbohydrate, and protein metabolism (Van den Berghe, 1991). However, the increasing prevalence of high-fat, calorie-dense diets has led to a surge in non-alcoholic fatty liver disease (NAFLD), a condition marked by excessive triglyceride accumulation in hepatocytes (Loomba et al., 2021). This lipid overload occurs when hepatic lipid synthesis or uptake exceeds the liver’s ability to oxidize or export lipids, ultimately leading to lipotoxicity and impaired cellular functions (Roumans et al., 2021). Clinically, NAFLD progresses through a spectrum that includes simple steatosis, non-alcoholic steatohepatitis (NASH), cirrhosis, and hepatocellular carcinoma (Seen et al., 2021; Pouwels et al., 2022). Epidemiological data has highlighted the alarming prevalence of NAFLD: 10%–20% in lean individuals, 65%–75% in the obese population, and up to 90% in morbidly obese individuals (Quek et al., 2023).

Piperine (PIP), a key alkaloid in black pepper (*Piper nigrum*) and long pepper (*Piper longum*), has a rich history in traditional medicine systems such as Ayurveda (Dludla et al., 2023). Modern pharmacological studies have confirmed its diverse therapeutic potential, including anti-inflammatory, antioxidant, anticancer, antimicrobial, and neuroprotective properties (Balakrishnan et al., 2023; Abdel-Daim et al., 2019; Wang et al., 2023). Notably, PIP has shown significant reduction in the levels of liver enzymes such as aspartate aminotransferase (AST) and alanine aminotransferase (ALT), as well as a decrease in serum triglycerides, cholesterol, and low-density lipoproteins (LDL), while enhancing high-density lipoprotein (HDL) levels (Nouri-Vaskeh et al., 2024; Hosseini et al., 2024). Furthermore, PIP has demonstrated the ability to mitigate hepatic steatosis and insulin resistance in high-fat diet-induced obesity models, primarily by modulating AMP-activated protein kinase (AMPK) signaling pathways (Wang et al., 2022).

Despite its notable pharmacological potential, piperine (PIP) remains clinically limited by its extremely poor aqueous solubility and extensive first-pass metabolism, both of which markedly diminish its oral bioavailability and therapeutic activity. These biopharmaceutical constraints necessitate advanced formulation strategies to optimize its pharmacokinetic profile. Several delivery platforms, including nanoemulsions (Alshehri et al., 2020), self-emulsifying drug delivery systems (SEDDS) (Xu et al., 2014), solid dispersions (Shao et al., 2018), and phospholipid (phytosome) complexes (Biswas et al., 2020), have been investigated to circumvent these limitations. Although these systems enhance the solubility and intestinal absorption of PIP, each presents inherent drawbacks: nanoemulsions and SEDDS may suffer from thermodynamic instability and surfactant-associated toxicity; solid dispersions, while improving dissolution, are susceptible to drug recrystallization and compromised storage stability; and phytosome complexes, despite improving membrane permeability, face challenges related to limited scalability. In this context, SLNs represent a particularly promising platform for the effective delivery of poorly water-soluble molecules such as PIP (Quijia et al., 2021). By integrating the advantages of liposomes and polymeric nanoparticles while mitigating their respective limitations, SLNs offer a biocompatible and biodegradable lipid matrix that reduce the risk of systemic accumulation and toxicity (Beloqui et al., 2016). In addition, SLNs effectively address the major biopharmaceutical barriers of PIP by improving its aqueous solubility, and thereby, oral bioavailability. Furthermore, their amenability to lyophilization enables the production of stable, free-flowing powders suitable for long-term storage (Mehnert and Mäder, 2012). Collectively, these attributes underscore the potential of SLNs as a superior delivery system for achieving targeted hepatic distribution and enhancing therapeutic outcomes in the management of non-alcoholic fatty liver disease (NAFLD).

In this study, SLNs were formulated to improve the hepatic delivery of PIP and assess its therapeutic efficacy in a high-fat diet-induced hyperlipidemia mouse model. Key biochemical markers of NAFLD, including glucose tolerance, ALT, AST, total cholesterol, and triglycerides, were evaluated. Moreover, liver histopathology was performed to validate the hepatoprotective effect of PIP-SLNs.

## 2 Materials and methods

### 2.1 Materials

Piperine (PIP), glycerol monostearate (GMS), stearic acid (SA), poloxamer 188, and poloxamer 407 were purchased from Sigma Aldrich, Bangalore, India. Hydrogenated soya phosphatidylcholine (HSPC) was obtained as a gift sample from Lipidome Life Sciences, Gujarat, India. Other chemical substances and reagents used in the research were of analytical grade.

### 2.2 Fourier transform infrared (FTIR) studies

Drug-lipid compatibility between the PIP and the lipids (GMS, SA, and HSPC) used for the preparation of solid lipid nanoparticles (SLNs) was analysed by FTIR spectrophotometer (Tensor 27, Bruker optics, Tokyo, Japan). The investigation entailed obtaining the IR spectra of PIP, and its physical mixture with lipids (Tang et al., 2017). The acquired peaks were compared to standards and interpreted to verify the possible existence of incompatibilities between the drug and lipids used for the manufacture of SLNs.

### 2.3 Formulation of piperine-loaded solid lipid nanoparticles (PIP-SLNs)

The PIP loaded SLNs were formulated using the hot homogenization method (Bustos Araya et al., 2025). Different lipids such as GMS, SA, and HSPC, and emulsifiers, namely, poloxamer 188 and poloxamer 407, were tried individually and in combination to obtain the optimized PIP-SLNs. Initially, a set of preparatory formulations were developed via a single lipid and a single emulsifier (Table 1). The procedure involved melting the lipid at 70 °C on a temperature controlled magnetic stirrer, approximately 10 °C above the melting point of the lipids. The PIP was added to the melted lipid to form a lipid-drug mixture. On the other hand, a defined amount of emulsifier was dispersed in 40 mL of deionized water and heated to 70 °C. Melted lipid-PIP mixture was then added to the hot emulsifier solution maintained at 70 °C with continuous stirring at 300 rpm for 3 h using a thermostat-controlled magnetic stirrer (IKA C-MAG HS4 digital, Germany). After 3 h of stirring, the hot mixture was subjected to high shear homogenization (IKA India Pvt. Ltd, Model-T25D, Germany) for 15 min at 7000 rpm to obtain an emulsion. This crude emulsion was subsequently sonicated for 10 min to get size-reduced PIP-SLNs. Finally, the PIP loaded SLNs was centrifuged for 30 min at 4500 rpm and the sediment was collected and frozen in liquid nitrogen. The frozen sediment was added with 5%w/v mannitol as cryo-protectant and lyophilized for 48 h at a 0.05 mmHg pressure using Labogene lyophilizer (CA, United States). The obtained 12 formulations were evaluated for percentage yield, particle size, Zeta potential and entrapment efficiency (%) and these data were analysed for final optimization (Table 1).

**TABLE 1:** Physico-chemical properties of preliminary formulations of PIP-SLNs.

Formula	GMS	SA	HSPC	P407	P188	Yield (%)	Entrapment efficiency (%)	Particle size (nm)
T1	358	--	--	--	210	39.2 ± 0.9	61.7 ± 1.3	655.1 ± 29.2
T2	537	--	--	--	210	29.7 ± 0.6	66.4 ± 0.8	728.2 ± 39.5
T3	358	--	--	350	--	36.6 ± 0.3	68.7 ± 1.1	347.8 ± 16.5
T4	537	--	--	350	--	27.1 ± 0.5	72.8 ± 2.1	418.2 ± 21.6
T5	--	284	--	--	210	57.8 ± 0.7	43.7 ± 1.8	561.7 ± 21.9
T6	--	426	--	--	210	44.5 ± 0.5	59.2 ± 0.6	628.7 ± 19.5
T7	--	284	--	350	--	61.9 ± 2.7	47.8 ± 0.9	284.7 ± 20.9
T8	--	426	--	350	--	47.6 ± 1.1	64.1 ± 2.3	331.9 ± 11.6
T9	--	--	76	--	210	67.7 ± 1.0	31.7 ± 1.7	698.7 ± 17.8
T10	--	--	114	--	210	54.1 ± 0.8	52.1 ± 0.8	757.4 ± 22.1
T11	--	--	76	350	--	69.5 ± 1.9	36.3 ± 0.6	253.2 ± 19.3
T12	--	--	114	350	--	51.6 ± 0.6	57.3 ± 2.9	309.5 ± 10.9

Data are represented as mean ± SD (n = 3).

### 2.4 Optimization of PIP-SLNs

Considering the properties of the preparatory formulations, such as percentage yield, particle size and entrapment efficiency, a set of PIP-SLNs were developed where two different lipids were combined in different molar ratios. In these formulations, lipid-1 (GMS) and lipid-2 (HSPC/SA) were used as mentioned in Table 2, and polaxamer-407 was employed as the emulsifier. The nanoparticles were prepared as above mentioned.

**TABLE 2:** Physicochemical properties of selected PIP-SLNs.

Formulation Code	GMS (mg)	SA (mg)	HSPC (mg)	P407 (mg)	Yield (%)	Entrapment efficiency (%)	Particle size (nm)
F1	358	--	76	350	59.5 ± 1.8	47.3 ± 1.4	480.0 ± 26.6
F2	537	--	76	350	53.12 ± 1.1	56.6 ± 2.5	1384.0 ± 10.2
F3	358	--	152	350	57.7 ± 0.9	50.9 ± 2.1	728.0 ± 44.3
F4	537	--	152	350	65.03 ± 2.1	60.3 ± 3.0	255.0 ± 27.0
F5	358	284	--	350	42.8 ± 3.6	61.5 ± 1.1	669.0 ± 26.7
F6	537	284	--	350	76.6 ± 3.8	72.3 ± 2.8	191.2 ± 27.9
F7	358	568	--	350	45.8 ± 1.5	57.7 ± 2.5	759.0 ± 10.2
F8	537	568	--	350	45.01 ± 1.9	65.3 ± 1.8	498.0 ± 26.8

Data are represented as mean ± SD (n = 3).

### 2.5 Characterization of optimized PIP-SLNs

#### 2.5.1 Formulation yield

PIP-SLNs were subjected to centrifugation at 4500 rpm at room temperature for 15 min. After centrifugation, the sediment was collected and dried at a temperature of 40 °C. The formulation yield (%) was calculated using the following equation (Vivek et al., 2007):

$$\%\text{ Yield}=\frac{\left(\text{Weight}\;\text{of}\;\text{SLNs}\;\text{obtained}\right)}{\left(\text{Weight}\;\text{of}\;\text{solid}\;\text{materials}\;\text{used}\;\text{in}\;\text{the}\;\text{formulation}\right)}x\;100$$

#### 2.5.2 Entrapment efficiency

The entrapment efficiency of PIP-SLNs was estimated by dissolving freeze dried PIP-SLNs in methanol/water mixture (80:20 v/v%). The mixture was passed through the 0.4 µm nylon syringe filter, diluted appropriately with the mobile phase, and sonicated for 10 min before being analysed by the HPLC instrument (Shimadzu Prominence-I, LC-2030C 3D Plus, Tokyo, Japan). Briefly, 10 µL sample solution was injected into C18 column (Phenomenex, 5 µ size, 150 mm length, 4.6 mm internal diameter) and the flow rate was maintained to1 mL/min. The samples were analysed at 343 nm using UV spectrophotometer photodiode detector. The entrapment efficiency (%) was calculated using the following formula (Gopalakrishna et al., 2023):

$$\%\text{ Entrapment efficiency}=\frac{\left(\text{Amount}\;\text{of}\;\text{PIP}\;\text{in}\;\text{SLNs}\right)}{\left(\text{Initial}\;\text{amount}\;\text{of}\;\text{PIP}\;\text{added}\right)}x\;100$$

#### 2.5.3 Particle size, polydispersity index, zeta potential measurement

The diameter, polydispersity index (PDI) and zeta potential of the PIP-SLNs was measured using a zeta sizer (Malvern Model- Nano ZS-90, Worcestershire, United Kingdom). 10 μL of each formulation was dispersed in 10 mL of deionized water, and sonicated for 10 min. The sonicated sample was collected in a cuvette and processed for size distribution measurement. The zeta potential of the PIP-SLNs was determined using an electrode cuvette. All measurements were conducted in triplicates.

#### 2.5.4 Surface morphology of PIP-SLNs

The surface morphology of the optimized formulation (F6) was studied by SEM analysis (Singh et al., 2022). The optimized formulation (F6) was photographed by scanning electron microscope (ZEISS ULTRA 55 Field emission scanning electron) at 100.00 KX magnification at 25 °C.

### 2.6 *In vitro* release studies

An *in vitro* release study for PIP from different PIP-SLNs was conducted by the dialysis method (Jamous et al., 2023). Briefly, the dried PIP-SLNs was dispersed in 5 mL of phosphate buffer (PBS; pH 7.4) and filled into a dialysis bag (cellulose tubing; Mwt cut off 12000–14000 Da). The dialysis bag was immersed in the receptor compartment containing 200 mL of PBS maintained at 37 °C ± 0.5 °C and stirred at a speed of 50 rpm. PIP content in the receptor compartment was measured at pre-determined time intervals (1, 2, 3, 4, 5, 6, 12, 24 and 48 h). Samples withdrawn from the receptor compartment at the specified time interval were sonicated, filtered through 0.4 µm syringe filter, quantified for PIP content by HPLC as aforementioned.

### 2.7 Pre-clinical studies

#### 2.7.1 Animals

Swiss albino male mice (25–30 g) were used in this study. All animals were housed under the temperature conditions of 21 °C–25 °C, and RH 60% ± 10% with a 12 h/12 h light/dark cycle. The study protocol was approved by the Institutional Animal Ethics Committee (IAEC) of Acharya and BM Reddy College of Pharmacy (Registration number: 997/C/06/CPCSEA). The study was performed in accordance with IAEC (IAEC/ABMRCP/2021- 22/2).

#### 2.7.2 Anti-hyperlipidaemic effect

The anti-hyperlipidemic activity of the optimized formulation (F6) was evaluated in comparison with pure piperine (PIP) and the reference standard silymarin. Animals were assigned to experimental groups using simple randomization to minimize selection bias. A total of five groups were established, each containing six animals (sample size determined based on previous studies reporting effect sizes for HFD-induced hyperlipidemia models and powered to detect a minimum difference of 20%–25% in lipid profile parameters with 80% power at α = 0.05). One group served as the normal control, and the remaining four groups received disease induction and/or treatment.

The normal control group received a standard laboratory diet, while all other groups were fed a high-fat diet (HFD) composed of regular chow supplemented with 5% cholesterol and 1% cholic acid, as described in earlier reports (Sampathkumar et al., 2011; Venkateshan et al., 2016). The study duration was 21 days, and treatment was administered once daily as per the following protocol:

Group 1 - Normal Control: Normal saline solution (per oral) + regular diet
Group 2 - Disease Control: High-fat diet only (Thirumalai et al., 2014)
Group 3 - Standard Treatment: HFD + silymarin (30 mg/kg, per oral)
Group 4 - Pure Drug Treatment: HFD + PIP (30 mg/kg, per oral)
Group 5 - Test Formulation (F6): HFD + F6 formulation (equivalent to 30 mg/kg PIP, per oral)

Throughout the study, animals were housed under identical environmental conditions, and treatment order was randomized daily to avoid handling-related bias. All outcome assessments were performed by investigators blinded to group allocation to ensure objective evaluation.

#### 2.7.3 Oral glucose tolerance test (OGTT)

The impact of treatments on glucose metabolism was estimated by oral glucose tolerance test (OGTT) (Andrikopoulos et al., 2008). Briefly, mice were deprived from food for overnight. Then the mice were randomly assigned to five groups: a normal control, a disease control, and test groups receiving silymarin, pure PIP, and an optimized formula (F6) of PIP-SLNs, respectively, as aforementioned. At 30 min post each treatment, 2 g/kg glucose solution was given orally, and blood glucose levels were measured using a glucometer prior to treatments (0 min), as well as 30 min and 60 min post glucose solution intake.

#### 2.7.4 Biochemical and histopathological studies

After 3 days of washout period post oral glucose tolerance test, blood specimens were collected via retro-orbital sinus puncture. This was followed by centrifugation at 4800 rpm for 30 min. The obtained serum was separated and stored at −20 °C. This serum sample was used for biochemical analysis of lipid profile (cholesterol and triglycerides) and liver function enzymes (alanine aminotransferase (ALT), and aspartate aminotransferase (AST) (Fabbrini et al., 2010), using the standard diagnostic kits.

At the end of the experiments and after blood sampling, animals were euthanized using diethyl ether and the liver was dissected. For histopathological examination, liver samples were first washed by PBS, then fixed in 10% formalin, dehydrated in ascending grades of alcohol, and paraffin embedding procedures were conducted. 5 μm thickness sections were then stained by haematoxylin-eosin (H & E) dyes and the sections were screened for signs of liver injury such as vesicular steatosis, lobular inflammation (lymphocytes, Kupffer cells) ballooning, Mallory-Denk bodies, fibrosis, and necrosis.

### 2.8 Statistical analysis

The experimental data was statistically evaluated using one-way ANOVA, followed by Tukey’s *post hoc* test. Results were presented as mean ± standard deviation (SD). Statistical analysis was performed using GraphPad Prism version 5 (GraphPad Software, Inc., 2010).

## 3 Result

### 3.1 Preparation of PIP- SLNs

The hot homogenization method was used for the formulation of PIP-SLNs. Preliminary formulations (T1 to T12) were developed by varying the lipid components and emulsifiers. Glyceryl monostearate (GMS), stearic acid (SA), and hydrogenated soy phosphatidylcholine (HSPC) were selected as the main lipid components based on their high solubility for PIP. Two emulsifiers, poloxamer 188 and poloxamer 407, were also evaluated. The preliminary formulations (T1 to T12) were evaluated for percent yield, entrapment efficiency, and particle size, aiming to develop SLNs with the highest possible yield and entrapment efficiency, while maintaining a small particle size.

### 3.2 Properties of PIP- SLNs

It was evident that, among the various tested lipids, SLNs prepared with GMS showed the highest entrapment efficiency. The percentage entrapment efficiency of SLNs prepared with GSM (T1 - T4) ranged from 61.7% ± 1.3% to 72.8% ± 2.1%. Whereas SLNs prepared with either SA or HSPC exhibited remarkably lower entrapment efficiencies ranging from 43.7% ± 1.8% to 64.1% ± 2.3% for SA-based SLNs and 31.7% ± 1.7% to 57.3% ± 2.9% for HSPC-based SLNs. Nonetheless, the percentage yield of SLNs prepared with GSM was much lower than those prepared with either SA or HSPC as the main lipid component. The % yield for SLNs prepared with either SA or HSPC reached 61.9% ± 2.7% (T7) and 69.5% ± 1.9% (T11), respectively, which were much higher than that observed with GMS-based SLNs (T1; 39.2% ± 0.9%). Furthermore, the particle size of SLNs prepared with either SA or HSPC were remarkably smaller than those prepared with GMS. The particle size of T7 and T11, prepared with SA and HSPC, respectively, was considerably lower than that of T3, prepared with GMS (284.7 ± 20.9 and 253.2 ± 19.3 vs. 347.8 ± 16.5 nm). Notably, regardless of the type of the lipid component, the particle size of all the prepared SLNs was found to be dependent on the type of emulsifier used. SLNs prepared with poloxamer 407 showed a significantly lower particle size compared to those prepared with poloxamer 188. The particle size of T4, prepared with poloxamer 407, was 418.2 ± 21.6 nm, which was significantly lower than that of T2 prepared with poloxamer 188 (728.2 ± 39.5 nm). Based on the obtained data, GMS, as the main lipid component, along with either SA or HSPC as a secondary lipid component and poloxamer 407 as emulsifier were further challenged for the formulation of PIP-SLNs in order to obtain an optimized formula having a maximum percentage yield, maximum entrapment efficiency along with smaller particle size.

Based on our preliminary experiments, a set of formulations (F1-F8) was developed using a blend of lipids (GMS/SA or GMS/HSPC) as lipid matrices and poloxamer 407 as the emulsifier (Table 2). The developed formulations were characterized for percentage yield, entrapment efficiency, particle size and the optimization goals were set to attain a formulation with a minimum vesicle size while having maximum percentage yield and maximum entrapment efficiency. It was evident that the percentage yield of all formulation remarkably increased upon using blend of lipids (GSM/SA or GS/HSPC) compared to SLNs prepared with GMS only. The percentage yield fluctuated from 42.8% ± 3.6% (F5) to 76.6% ± 3.8% (F6) (Table 2). In addition, the lipid matrix composition was found to affect the extent of drug entrapment within solid lipid nanoparticles. As summarized in Table 2, the entrapment efficiency of various PIP-SLNs was estimated to range from 47.3% ± 1.4% (F1) to 72.3% ± 2.8% (F6) (Table 2). It was observed that, SLNs prepared GMS/SA as lipid matrices showed higher entrapment efficiency than those prepared with GMS/HSPC. Most importantly, the particle size of the formulated PIP-SLNs ranged from 191.2 ± 27.9 nm (F6) to 1384% ± 10.2% (F2). The zeta potential of the formulations ranged from −0.06 ± 5.07 (F3) to −27.3 ± 4.55 (F8), varying according to the specific formulation and lipid composition (Figure 2d).

### 3.3 Formulation compatibility

**FIGURE 1:**
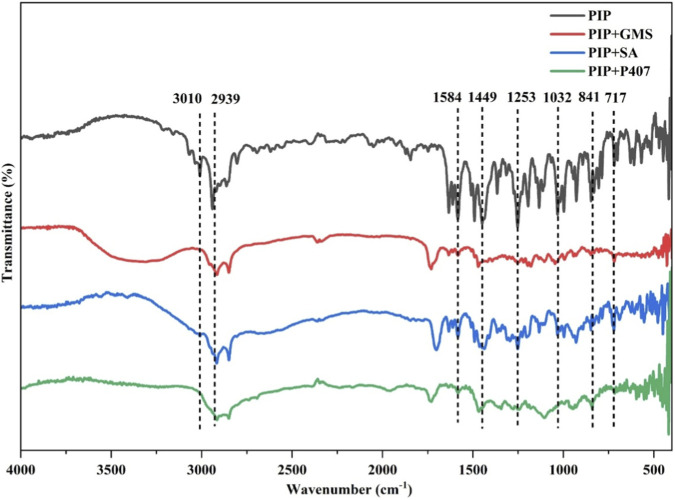
FTIR spectra depict transmittance as a function of wave number for PIP, PIP+GMS, PIP+SA, and PIP+P407, with characteristic bands identified at 3010, 2939, 1584, 1449, 1253, 1032, 841, and 717 cm^−1^.

Fourier-transform infrared (FTIR) spectroscopy was performed to assess the compatibility between PIP and the lipid components selected for the formulation of PIP-SLNs. The spectrum of pure PIP exhibited distinct characteristic peaks: aliphatic C-H stretching at 2939-3010 cm^−1^, a strong C=O stretching at 1583 cm^−1^, C=C stretching at 1449 cm^−1^, C-H bending vibrations of the aromatic (841 cm^−1^) and substituted aromatic ring (717 cm^−1^), and C-O stretching at 1253 cm^−1^. These key functional group peaks were also clearly observed in the spectra of the physical mixtures of PIP with the selected lipid components. No significant shifts, disappearance, or emergence of new peaks were noted in the physical mixtures (Figure 1).

### 3.4 Characterization of optimized PIP-SLNs

Among the formulations, F6 demonstrated the most favorable combination of properties; highest percentage yield (76.6% ± 3.8%), maximum entrapment efficiency (72.3% ± 2.81%), and smallest particle size (191.2 ± 27.9 nm), favorable zeta potential (−20.2 ± 1.3) and therefore was selected for further investigations.

#### 3.4.1 Surface charge and morphology

**FIGURE 2:**
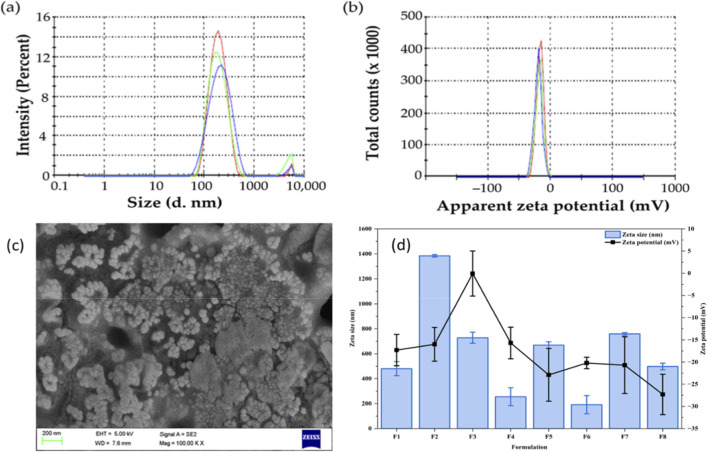
**(a)** Particle size distribution profile of PIP-SLNs, **(b)** zeta potential distribution profile, **(c)** Scanning Electron Micrograph of the optimized PIP-SLNs formulation (F6) showing nanoscale morphology, and **(d)** comparative particle size and zeta potential profiles of formulations F1–F8 (data expressed as mean ± SD, n = 3).

The optimized PIP-SLNs (F6) demonstrated an average particle size of 191.2 ± 27.9 nm (Figure 2a), and the PDI was found to be 0.28. The zeta potential of the optimized formulation was measured at −20.2 ± 1.3 mV (Figure 2b), reflecting the surface charge and colloidal stability of the nanoparticles. The surface morphology of the optimized PIP-SLNs (F6) when examined using scanning electron microscopy (SEM) revealed spherical particles with smooth surfaces, although some degree of agglomeration was observed among the nanoparticles (Figure 2c).

#### 3.4.2 *In vitro* release

**FIGURE 3:**
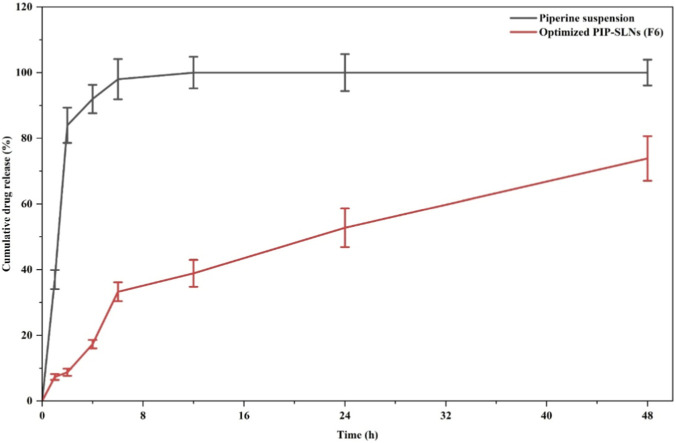
Cumulative PIP release profile over time, comparing PIP suspension and optimized PIP-SLNs (F6). The PIP suspension exhibits rapid release, reaching approximately 100% drug release within 8 hours, whereas the optimized PIP-SLNs (F6) demonstrate a sustained release pattern, achieving around 40% cumulative release over 48 hours (data expressed as mean ± SD, n = 3).

The *in vitro* release study showed that PIP-SLNs (F6) exhibited a biphasic release pattern, with approximately 18% of Piperine released within the first 4 h and a cumulative release of about 70% over 48 h (Figure 3). In contrast, the piperine suspension released nearly 85% of the drug within the first 2 h.

Kinetic analysis demonstrated that the release from PIP-SLNs corresponded closely with the Higuchi model (R^2^ = 0.976), followed by Zero-order kinetics (R^2^ = 0.877). The Korsmeyer-Peppas model yielded an exponent of n = 0.63 (R^2^ = 0.95). For the Piperine suspension, the release data showed the highest correlation with the First-order model (R^2^ = 0.969), while correlations with Zero-order, Higuchi, and Korsmeyer-Peppas models were comparatively low.

### 3.5 Preclinical studies

#### 3.5.1 Effects of PIP-SLNs on oral glucose tolerance test

**FIGURE 4:**
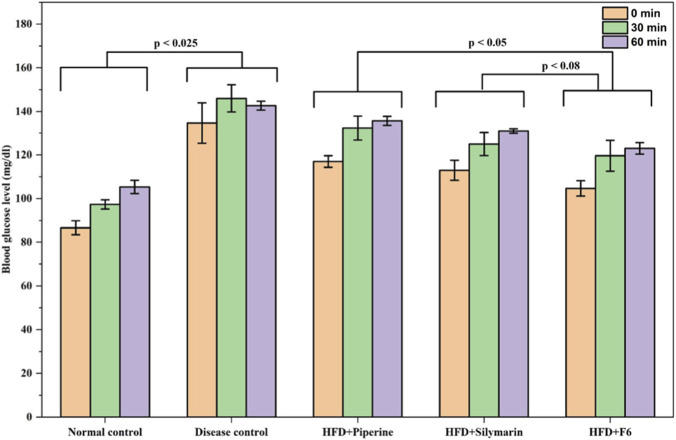
Hypoglycemic effects of PIP formulations in high-fat diet (HFD)-induced mice model. Blood glucose levels (mg/dL) across normal control, disease control, HFD + PIP, HFD + silymarin, and HFD + F6 groups at 0, 30, and 60 minutes post-administration. HFD groups show elevated levels versus normal control. Significant differences are indicated with p-values of less than 0.025, 0.05, and 0.08 (data expressed as mean ± SD, n = 6).

The oral glucose tolerance test (OGTT) was performed to understand the impact of PIP-SLNs on hepatic glucose metabolism. The glucose tolerance profiles are presented in Figure 4. The HFD-fed mice (disease control) exhibited significantly elevated blood glucose levels at all time points compared to the normal control group (*p* < 0.025).

Treatment with pure PIP (group 4) and silymarin (group 3) resulted in a significant reduction in blood glucose levels, compared to the disease control. Notably, mice treated with the optimized PIP-SLNs formulation (F6) showed a substantial decline in glucose levels by the end of 60 min. Glucose utilization in the formulation treated group (F6) was closely similar to silymarin (P < 0.05) and better than pure PIP (P < 0.08), indicating the improved therapeutic potential of PIP-SLNs. These findings suggest higher glucose utilization in the F6 treated mice.

#### 3.5.2 Effects of PIP- SLNs on serum lipids

**FIGURE 5:**
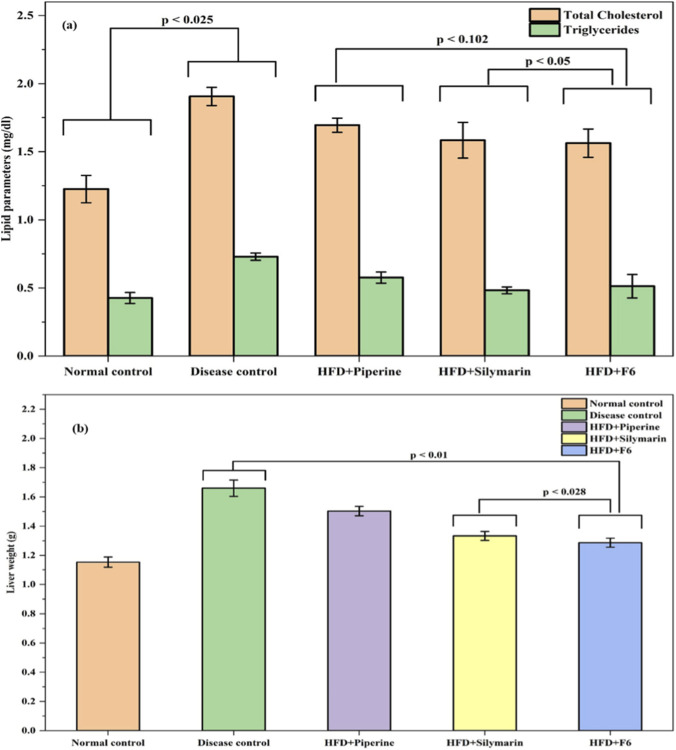
**(a)** Hypolipidemic effects on total cholesterol and triglycerides, and **(b)** changes in liver weight across groups; normal control, disease control, HFD + PIP, HFD + silymarin, and HFD + F6 in high-fat diet (HFD) induced mice. Significant differences indicated by p-values (data expressed as mean ± SD, n = 6).

The anti-hyperlipidemic potential of the optimized PIP-SLNs (F6) was evaluated on a high-fat diet (HFD) induced hyperlipidemia mice model (Du et al., 2020). Acute exposure to HFD, significantly elevated serum total cholesterol (TC) and triglyceride (TG) levels compared to the normal control group (*p* < 0.025) (Figure 5a).

Treatment with the optimized PIP-SLNs (F6) effectively reduced both TC and TG levels, restoring them to near-normal values. The lipid-lowering activity of F6 was on par with the standard silymarin treatment (p < 0.05), and it outperformed pure piperine in restoring lipid levels (p < 0.102 vs. F6). (Figure 5a).

In addition, liver weight significantly increased in HFD fed mice, compared to the controls (Figure 5b). Treatment with pure PIP showed minimal improvement, while the optimized SLNs formulation (F6) significantly reduced liver weight to near normal levels. The hepatoprotective effect of F6 was observed to be equivalent to that of silymarin (*p* < 0.028) (Figure 5b).

#### 3.5.3 Effects of PIP-SLNs on hepatic enzymes serum levels

**FIGURE 6:**
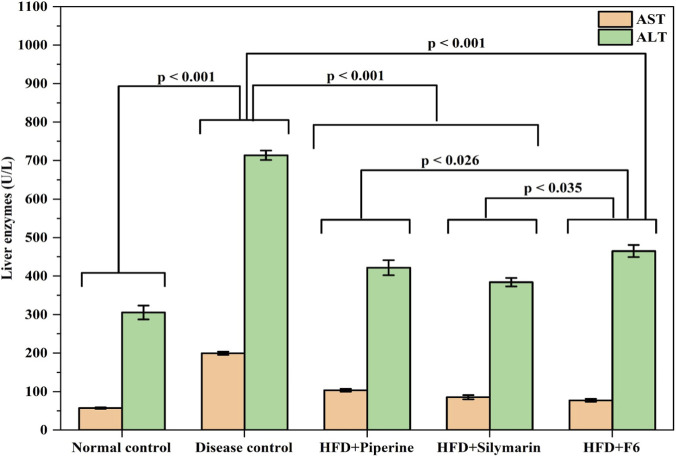
Levels of alanine aminotransferase (ALT) and aspartate aminotransferase (AST) across groups; normal control, disease control, HFD + PIP, HFD + silymarin, and HFD + F6 in high-fat diet (HFD) induced mice, with ALT significantly elevated over AST and highest in disease control; statistical significance indicated by p-values (data expressed as mean ± SD, n = 6).

The hepatoprotective activity of the optimized PIP-SLNs (F6) was assessed by determining the serum levels of liver function biomarkers, alanine aminotransferase (ALT) and aspartate aminotransferase (AST). As shown in Figure 6, the disease control group (HFD-fed mice) exhibited a significant elevation in ALT and AST levels, compared to the normal control group (*p* < 0.001), indicating hepatic injury.

Treatment of disease control group with pure PIP or silymarin significantly reduced serum ALT and AST levels (*p* < 0.001 vs. disease control). Notably, the group treated with optimized PIP-SLNs (F6) showed marked improvement, with enzyme levels nearly returning to those of the normal control. This reduction was statistically significant compared to the disease group (*p* < 0.001), and also equivalent to the silymarin group (*p* < 0.035) and better compared to pure piperine group (*p* < 0.026).

#### 3.5.4 Histopathology studies

**FIGURE 7:**
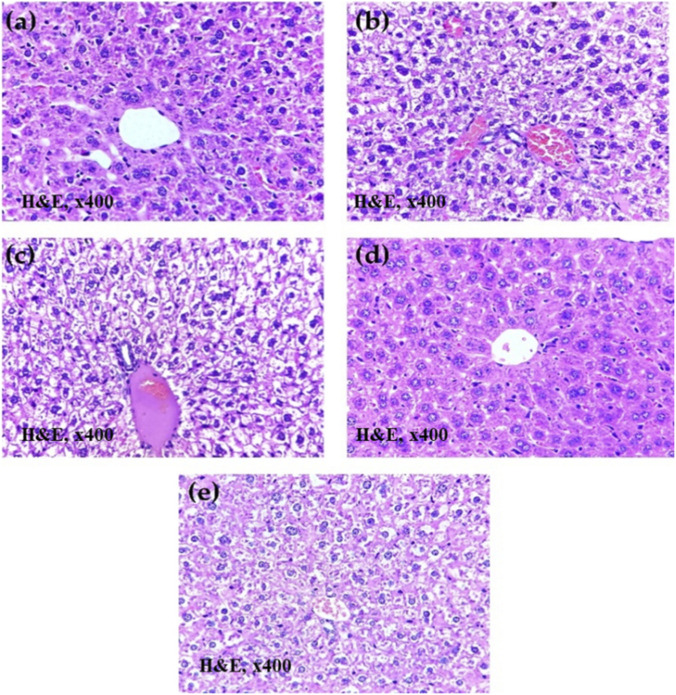
Histopathology of liver sections (H&E, 400×) from **(a)** normal control, **(b)** HFD-fed disease control, **(c)** HFD + PIP suspension, **(d)** HFD + silymarin (standard drug), and **(e)** HFD + optimized PIP-SLNs (F6) groups, revealing variations in cellular density, nuclear morphology, cytoplasmic features, and vascular structures.

The protective effects of PIP-SLNs on hepatic architecture and liver structural integrity were evaluated through histopathological analysis (Figure 7). In the normal control group (Figure 7a), liver sections displayed intact parenchymal architecture with well-preserved hepatocytes and sinusoids. In contrast, the disease control group (Figure 7b) showed severe hepatocellular damage, characterized by diffuse cytoplasmic vacuolation of hepatocytes and congested blood vessels. The pure PIP treated group (Figure 7c) exhibited moderate hepatocellular degeneration, including cytoplasmic vacuolation and blood vessel congestion. The silymarin-treated group (Figure 7d) demonstrated largely preserved hepatic structure with intact hepatocytes and sinusoids. Notably, the group treated with the optimized PIP-SLNs (F6) (Figure 7e) exhibited mild hepatocellular alterations, with mostly intact sinusoids and only a few congested vessels.

## 4 Discussion

### 4.1 Preparation and optimization of PIP- SLNs

In this study, a stepwise screening strategy was employed to guide the preliminary formulation development. In the initial phase, twelve trial formulations (Table 1) were prepared to examine the effects of varying lipid matrices (GMS, SA, and HSPC) and surfactants (poloxamer 407 and poloxamer 188) on the successful formation of SLNs. Each formulation was evaluated in terms of production yield, entrapment efficiency, and particle size, which were used as practical indicators of formulation feasibility and physical stability. The initial screening results highlighted that both the lipid type and emulsifier selection had marked effects on production yield, entrapment efficiency, and particle size of PIP-loaded SLNs.

Among the tested lipids, GMS resulted in the highest drug entrapment, likely attributable to its favorable solubilizing capacity and compatibility with PIP. GMS, a semi-solid lipid with a melting point near 60 °C, provides a structurally stable matrix that supports efficient drug incorporation. However, its relatively high viscosity may impede droplet disruption during homogenization (El-Emam et al., 2021), which can reduce nanoparticle formation efficiency. This was reflected by the lower yields observed for some GMS-containing formulations, such as T1 (39.2% ± 0.9%) and T3 (36.6% ± 0.3%) (Table 1). In contrast, SLNs incorporating SA or HSPC produced higher yields and relatively smaller particle sizes. SA, with a relatively lower viscosity and higher fluidity at room temperature compared to GMS (Wang et al., 2025), facilitated more efficient dispersion during homogenization, as manifested by higher yields of formulations T5 (57.8% ± 0.7%) and T7 (61.9% ± 2.7%). Similarly, HSPC is known to provide structural stability to the lipid matrix and improve emulsification, promoting formation of more uniform particles with an increasing overall yield (T9 67.7% ± 1.0% and T11 69.5% ± 1.9%). Nevertheless, both SA and HSPC containing formulations displayed slightly lower entrapment efficiencies than GMS based systems, however, their improved yields and particle-size profiles supported their plausible use as co-lipids to enhance SLNs production efficiency.

The effect of emulsifier type was also investigated. Formulations stabilized with poloxamer 407 consistently produced smaller particle sizes than those using poloxamer 188. The enhanced emulsification capacity of poloxamer 407 is attributed to its block-copolymer structure, which confers efficient reduction of interfacial tension during homogenization and promotes formation of finely dispersed droplets (Krstonošić et al., 2024). This behavior was reflected in the markedly smaller particle sizes in poloxamer 407 formulations (T3 347.8 ± 16.5 nm, T7 284.7 ± 20.9 nm) relative to their poloxamer 188 counterparts (T1 655.1 ± 29.2 nm, T5 561.7 ± 21.9 nm). Given that achieving smaller particle sizes is particularly advantageous for improving drug stability and dissolution, poloxamer 407 was selected as the main emulsifier for further SLNs formulation.

Based on the outcomes of the initial formulation trials, optimization efforts focused on selecting the best lipid combinations and emulsifier in order to maximize production yield, entrapment efficiency, and control particle size. Eight formulations composed of GMS as the principal lipid, combined with either SA or HSPC as co-lipids, while poloxamer 407 served as the stabilizing emulsifier were prepared. These formulations demonstrated notable improvements in all the tested evaluation parameters. In terms of entrapment efficiency, the GMS/SA formulation (F6) performed better than the GMS/HSPC formulation (F3). This is likely due to the solubility of PIP in the SA-rich lipid phase, which may facilitate more efficient drug incorporation into the lipid core (Zhang et al., 2023). Furthermore, the fabricated SLNs exhibited particle sizes ranging from 191.2 ± 27.9 nm (F6) to 1384% ± 10.2% (F2), emphasizing their potential to effectively target the liver (Gaumet et al., 2008). Of particular, formulation F6, which employed a GMS/SA binary lipid system, achieved the highest production yield (76.6% ± 3.8%) and entrapment efficiency (72.3% ± 2.8%), alongside the smallest mean particle size (191.2 ± 27.9 nm), zeta potential −20.2 ± 1.3 mV and accordingly was identified as the optimized formula and was used for further investigations.

### 4.2 Characterization of optimized PIP-SLNs

#### 4.2.1 Drug-lipid compatibility

Drug-excipients compatibility is essential for ensuring formulation stability and promoting efficient drug incorporation, while minimizing the risk of drug degradation or loss of bioactivity during processing and storage. Herein, FTIR analysis provided clear evidence supporting the compatibility of the selected lipid excipients with PIP and their suitability for formulating stable SLNs. The characteristic absorption bands of PIP, including the aliphatic C–H stretching (2939-2860 cm^−1^), C=O stretching (∼1583 cm^−1^), the C=C stretching (∼1449 cm^−1^), and the C–O stretching (∼1253 cm^−1^), C-H bending vibrations of the aromatic (841 cm^−1^) and substituted aromatic ring (717 cm^−1^) were distinctly preserved in the spectra of physical mixtures containing different lipids. The retention of these signature peaks without marked shifts, alterations in intensity, disappearance of original bands, or formation of new peaks suggests the absence of chemical incompatibilities or strong intermolecular interactions such as covalent bonding, esterification, or oxidative degradation.

#### 4.2.2 Surface charge and morphology

The physicochemical characteristics of the F6 formulation, suggested that it was well-suited for hepatic delivery. The particle size of approximately 191 nm lied within the optimal range for passive targeting of the liver, allowing efficient passage through fenestrated hepatic capillaries (Abu Lila et al., 2024). This nano-size also supported enhanced bioavailability and improved systemic circulation. The PDI value of 0.28 indicated a uniform particle size distribution. Since PDI values below 0.3 are generally considered acceptable for colloidal stability, this result reflected the formulation’s homogeneity, and reproducibility during processing.

Zeta potential plays a vital role in predicting the stability of nanosuspensions. Nanoparticles with high surface charge-whether positive or negative, tend to repel one another, thereby preventing aggregation (Abu Lila et al., 2024). Zeta potential of −20.2 ± 1.3 mV was observed for F6 (Figure 2b) which suggested moderate electrostatic repulsion among particles and this, when combined with low PDI, contributed to the physical stability of the formulation. Although higher absolute zeta potential values (e.g., >30 mV) are generally associated with enhanced stability, a zeta potential of −20.2 ± 1.3 mV, particularly in conjunction with a stabilizing emulsifier such as poloxamer 407, can be sufficient to maintain colloidal dispersion over time.

SEM analysis provided valuable insights into the topographical characteristics of the optimized PIP-SLNs. The spherical shape and smooth surface morphology of the particles suggested successful formulation using the hot homogenization technique (Figure 2c). Generally spherical nanoparticles are preferred for drug delivery applications, as they are associated with better cellular uptake, and uniform drug distribution. The agglomeration of the particles in the lyophilized sample is not uncommon and may be attributed to the absence of a cryoprotectant during the freeze-drying process or residual moisture content (Almalik et al., 2017). However, such agglomeration typically does not impact re-dispersibility or drug release if the formulation readily reconstitutes in aqueous media. The overall morphology supported the previously observed favorable particle size and homogeneity (PDI), affirming the quality and integrity of the optimized SLNs. Overall, these findings confirmed that the optimized PIP-SLNs possess favorable characteristics, for further *in vivo* and pharmacological evaluation.

### 4.3 *In vitro* release

A comparative assessment of the release behaviour of the Piperine suspension and PIP-SLNs (F6) highlights clear distinctions in their mechanistic profiles and therapeutic suitability for hepatic delivery. PIP-SLNs exhibited a characteristic biphasic release behavior typical of lipid-based nanoparticles, beginning with a mild initial burst attributable to surface-associated Piperine, followed by a sustained-release phase associated with drug diffusion through the lipid matrix. This behavior aligns with earlier findings on lipidic nanostructures, where hydrophobic drug-lipid interactions slow release and promote prolonged drug availability (Ghasemiyeh and Mohammadi-Samani, 2018; Osanlou et al., 2022). Kinetic modelling further supports the controlled-release characteristics of PIP-SLNs, as evidenced by their strong alignment with zero-order kinetics (R^2^ = 0.877), indicating concentration-independent release, and an excellent Higuchi fit (R^2^ = 0.976), confirming diffusion-dominated transport through the lipid core. The Korsmeyer-Peppas exponent (n = 0.63; R^2^ = 0.95) additionally reflects anomalous transport, combining diffusion and matrix relaxation an advantageous mechanism for sustained hepatic exposure (Sateesha et al., 2010).

In contrast, the Piperine suspension exhibited a rapid, dissolution-driven release with near-complete early-phase drug liberation. Its poor fit to zero-order (R^2^ = 0.24), Higuchi (R^2^ = 0.49), and Korsmeyer-Peppas models (n = 0.19; R^2^ = 0.52) and strong fit to the first-order model (R^2^ = 0.969) indicate a burst, concentration-dependent release pattern that is unsuitable for hepatic circulation, where sustained exposure is essential to enhance hepatocellular uptake and reduce rapid systemic clearance. Overall, the biphasic, diffusion-controlled, and sustained release behavior of PIP-SLNs (F6) confirms their capability to provide prolonged and regulated drug release, a characteristic essential for achieving effective hepatic circulation. Such controlled release is particularly advantageous for poorly water-soluble drugs like Piperine, supporting improved pharmacokinetic and therapeutic outcomes.

### 4.4 Preclinical studies

#### 4.4.1 Effects of PIP-SLNs on oral glucose tolerance test

The OGTT results demonstrate the potential of PIP-SLNs to significantly improve glucose utilization and regulate blood glucose levels in high-fat diet-induced hyperglycemic mice. Elevated glucose levels in the HFD group confirm impaired glucose metabolism, a common manifestation of metabolic stress and insulin resistance. The glucose-lowering effect observed in the F6treated group was not only statistically significant but also biologically relevant, as it closely approached the glucose levels of the normal control group across all tested time points. This effect was superior to that of pure PIP, and equivalent to silymarin, indicating that the nanoparticle-based formulation enhanced the bioavailability, and the therapeutic efficacy of PIP.

The observed outcomes may be attributed to improved hepatic targeting and controlled release characteristics of SLNs, which potentially enhanced drug accumulation in the liver, the primary site for glucose uptake and metabolism. The results also affirmed the role of SLNs as effective delivery carriers for hydrophobic bioactive like PIP, enabling sustained action and better therapeutic outcomes in glucose regulation. Thus, the findings collectively supported the potential of optimized PIP-SLNs as a promising formulation approach for managing glucose dysregulation in metabolic disorders, such as diabetes and non-alcoholic fatty liver disease.

#### 4.4.2 Effects of PIP-SLNs on serum lipids

Excessive fat accumulation from high-fat dietary intake led to dyslipidemia and hepatic steatosis, hallmark features of metabolic disorders such as non-alcoholic fatty liver disease (NAFLD) (Elnaggar et al., 2015). The current study demonstrated the potent anti-hyperlipidemic and hepatoprotective effects of the optimized PIP-SLNs (F6) in an HFD-mice model. The significant reduction in serum TC and TG levels following F6 treatment, implied enhanced lipid-lowering activity, which was comparable to the standard silymarin and superior to pure PIP (Figure 5a). These findings favored the notion that encapsulation of PIP in SLNs enhanced its bioavailability, stability, and therapeutic efficacy, likely through improved hepatic delivery and prolonged circulation. Additionally, the ability of F6 to restore liver weight to normal levels suggested that it protects against hepatic lipid accumulation and structural damage. Interestingly, this effect surpassed that of silymarin, highlighting the enhanced hepatoprotective potential of the nano-formulated PIP (Figure 5b). The superior performance of F6 over pure PIP also underlines the benefit of SLNs in overcoming the limitations of hydrophobic drugs, such as poor solubility and rapid metabolism. Collectively, these results reinforced the therapeutic usage of PIP-SLNs based delivery systems, in managing HFD induced metabolic dysfunctions, including hyperlipidemia and fatty liver-associated hepatomegaly.

#### 4.4.3 Effects of PIP-SLNs on hepatic enzymes serum levels

Serum aminotransferases: aspartate aminotransferase (AST), and alanine aminotransferase (ALT), are essential enzymes that aid in carbohydrate and amino acid metabolism (Fabbrini et al., 2010). Elevated aminotransferases, particularly ALT and AST, are hallmark indicators of hepatocellular damage in metabolic liver diseases such as in NAFLD. The marked rise in these enzymes in HFD-fed mice confirms the onset of hepatic dysfunction in this model.

Treatment with pure PIP or silymarin moderately reduced enzyme levels, reflecting some degree of hepatoprotection. However, the optimized PIP-SLNs (F6) demonstrated a notable protective effect, efficiently restoring ALT and AST levels to near normal values (Figure 6). This pronounced effect highlighted the potential of SLNs in enhancing drug delivery to hepatic tissues (Mishra et al., 2018), likely due to improved solubility, bioavailability, and sustained release of PIP.

The significant difference in efficacy between F6 and both pure PIP and silymarin further reinforced the hypothesis that nanoencapsulation improved the therapeutic outcomes, especially in conditions like NAFLD that require targeted hepatic intervention (Ashfaq et al., 2023; Guo et al., 2016). These findings confirmed the role of PIP-SLNs (F6) as a promising hepatoprotective agent in the context of diet-induced liver injury.

#### 4.4.4 Histopathology studies

Histopathological examination provided critical insight into the tissue level damage, and recovery associated with liver injury. In this study, the high fat diet (HFD) induced marked hepatic damage, evident from diffuse hepatocyte vacuolation, and vascular congestion, in the disease control group (Baz et al., 2022); thus, reflecting the pathological features of non-alcoholic fatty liver disease (NAFLD). Treatment with pure PIP showed only partial protection, as evidenced by moderate hepatocellular injury. Conversely, the silymarin-treated group exhibited considerable protection with largely preserved hepatic architecture, supporting its established role as a hepatoprotective agent. Importantly, animals treated with the optimized PIP-SLNs (F6) demonstrated a substantial histological improvement. The presence of only mild hepatocyte vacuolation, and largely intact sinusoids suggested effective hepatoprotection (Yang et al., 2017). These findings indicated that the SLN-based delivery system enhanced hepatic accumulation of PIP, contributing to improved anti-hyperlipidemic and hepatoprotective outcomes compared to pure drug administration.

Comparable outcomes have been reported for curcumin and resveratrol based nano therapies. Curcumin nanoparticles have been shown to lessen hepatic steatosis and inflammatory responses by improving bioavailability and directing drug delivery to target tissues (Jazayeri-Tehrani et al., 2019). Likewise, nano-resveratrol systems have demonstrated benefits by mitigating oxidative stress and modulating lipid metabolism through sustained release and enhanced hepatic uptake (Teng et al., 2019). In this context, the notable hepatoprotective effects achieved with PIP-SLNs such as decreased steatosis, reduced vacuolar changes, and improved biochemical markers indicate that nano-encapsulated piperine (PIP-SLNs) may offer therapeutic benefits comparable to these established nano-formulations, underscoring the need for future direct comparative evaluations.

## 5 Conclusion

This study successfully developed and characterized piperine loaded solid lipid nanoparticles (PIP-SLNs) to enhance the oral bioavailability and hepatoprotective efficacy of piperine in non-alcoholic fatty liver disease (NAFLD). The optimized formulation (F6) demonstrated desirable physicochemical features, including nanoscale size, appropriate zeta potential, high entrapment efficiency, spherical morphology, and sustained drug release up to 48 h, indicating improved stability and prolonged circulation. *In vivo* studies in hyperlipidemic Swiss albino mice showed that PIP-SLNs produced significant reductions (p < 0.05) in blood glucose, hepatic enzymes (AST and ALT), total cholesterol, triglycerides, and liver weight, outperforming plain piperine suspension. Histopathological findings further confirmed notable restoration of liver architecture, supporting the enhanced therapeutic effectiveness of the nano-formulation. Despite these promising outcomes, the research is limited by its reliance on a single animal model, short treatment duration, and the absence of detailed mechanistic or pharmacokinetic investigations. Future studies should incorporate molecular analyses, extended safety evaluations, and pharmacokinetic profiling to better elucidate the therapeutic pathways and long-term effects. From a translational standpoint, process scalability, long-term formulation stability, and clinical validation in human subjects represent crucial next steps. Overall, PIP-SLNs offer a compelling platform for advancing piperine as a viable therapeutic option for NAFLD.

## Data Availability

The original contributions presented in the study are included in the article/supplementary material, further inquiries can be directed to the corresponding author.
